# Factors Influencing the Sex Ratio at Birth in India: A New Analysis based on Births Occurring between 2005 and 2016

**DOI:** 10.1111/sifp.12147

**Published:** 2021-02-22

**Authors:** Abhishek Singh, Kaushalendra Kumar, Ajit Kumar Yadav, K. S. James, Lotus McDougal, Yamini Atmavilas, Anita Raj

**Affiliations:** ^1^ Abhishek Singh is Professor Department of Public Health & Mortality Studies International Institute for Population Sciences Govandi Station Road, Deonar Mumbai 400088 India; ^2^ Kaushalendra Kumar is Assistant Professor Department of Public Health & Mortality Studies International Institute for Population Sciences Govandi Station Road, Deonar Mumbai 400088 India; ^3^ Ajit Kumar Yadav is Research Analyst GENDER Project International Institute for Population Sciences Govandi Station Road, Deonar Mumbai 400088 India; ^4^ K. S. James is Director and Senior Professor International Institute for Population Sciences Govandi Station Road, Deonar Mumbai 400088 India; ^5^ Lotus McDougal is Associate Project Scientist Center on Gender Equity and Health University of California San Diego San Diego CA; ^6^ Yamini Atmavilas is Senior Programme Officer Bill and Melinda Gates Foundation New Delhi India; ^7^ Anita Raj is Tata Chancellor Professor of Medicine and Director Center on Gender Equity and Health University of California San Diego San Diego CA

## Abstract

Previous research on sex ratio at birth (SRB) in India has largely relied on macro‐analysis of census data that do not contain the breadth of factors needed to explain patterns in SRB. Additionally, no previous research has examined the differentiation of factors associated with SRB across birth orders, a key determinant in societies affected by son preference. This study aims to fill these gaps using micro‐data related to 553,461 births occurring between 2005 and 2016 collected as part of the 2015–2016 National Family Health Survey. Analyses used multivariable logistic regressions stratified by birth order to examine associations with SRB at the national level. The SRB at birth order 1 was outside the biological normal limit, and generally increased with birth order. First births in households with wealth in the middle and richest quintiles, with mothers who desired a higher ideal number of sons than daughters, and in lower fertility communities had a higher probability of being male. Most SRB correlates were visible at birth orders 3 or higher. Programs and policies designed to address India's male‐skewed SRB must consider the diverse factors that influence SRB, particularly for higher order births.

## INTRODUCTION

Concerns regarding sex ratio imbalance among children in India are decades old. This discussion acquired prominence during the early 1990s with the publication of the landmark article “More than 100 million women are missing” by Nobel Laureate Amartya Sen (1990), highlighting the issue of sex selective abortions and the preference for sons across Asia in general, and India in particular. Other scientific literature has established that India and China account for most of the world's female deficit (Bongaarts and Guilmoto 2015; Chao et al. 2019; Guilmoto et al. 2018; Klasen and Wink 2002), and that the sex ratio of children aged zero to six years (CSR) in India has long been in favor of males (Arokiasamy 2007; Arokiasamy and Goli 2012; Bhat and Zavier 2007; Mishra et al. 2009; Office of Registrar General of India 2011; Premi 2001). According to the most recent Indian census, the CSR was 109 males per 100 females (Office of Registrar General of India 2011). More worrisome is the fact that the CSR increased from 106 in 1991 to 108 in 2001 and further to 109 in 2011 (Jha et al. 2011; Rajan et al. 2015). Proximally, these high CSRs are attributable to some combination of high sex ratio at birth (SRB) and excess female mortality during childhood (Alkema et al. 2014; Arnold et al. 2002; DasGupta and Bhat 1997; Diamond‐Smith et al. 2019; Guilmoto et al. 2018; Mishra et al. 2009; Sudha and Rajan 1999; Visaria 1971). Despite indications from time series data from the Indian Sample Registration System (SRS) that the sex differential in mortality has narrowed in recent years (Registrar General of India 2015), the CSR has been rising steadily in India. In a scenario in which the sex differentials in mortality are rapidly diminishing and there is no sign of an increase in the excess female under‐5 deaths (Alkema et al. 2014; Bongaarts and Guilmoto 2015; Hill and Upchurch 1995), the rising trend in CSR can only be explained by high SRB.

**FIGURE 1:**
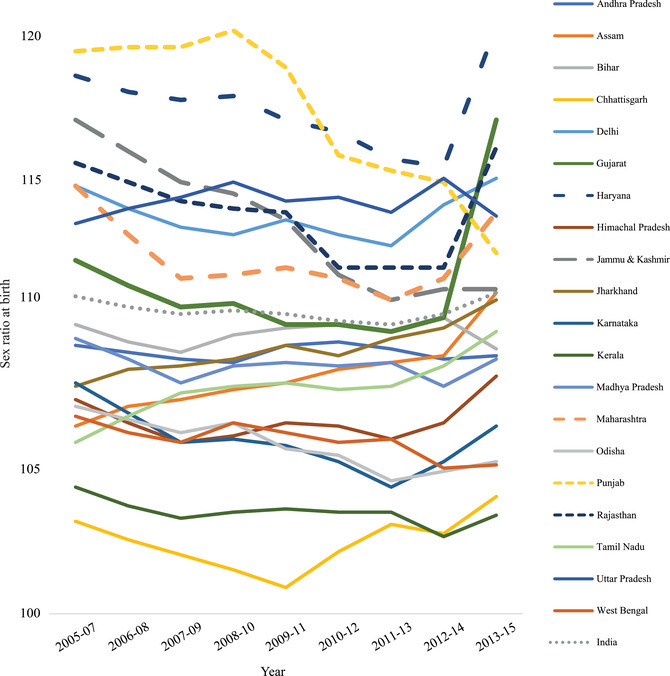
Trends in SRB in India and selected states, 2005–2015SOURCE: Sample Registration System, India.

Under normal circumstances, SRB varies between 103 and 106 male births per 100 female births (United Nations Secretariat 1998; Visaria 1971), with the estimated global average value being 105 male births per 100 female births (Bongaarts 2013; Dyson 2012). However, recent data from India's SRS indicate a SRB of 111 males per 100 females in 2015, with levels over the last 10 years varying between 110 and 112 (Office of the Registrar General of India 2016). The estimate of India's SRB for 2017 by Chao and Yadav (2019) was 110. Moreover, a considerable number of Indian states have a SRB higher than the national average (Figure 1) (Chao and Yadav 2019). Researchers mostly attribute the high SRB to incidents of sex selective abortion in regions where son preference remains strong (Arnold et al. 2002; Bongaarts 2013; DasGupta and Bhat 1997; Diamond‐Smith et al. 2019; Roy and Chattopadhyay 2012; Sudha and Rajan 1999), though there is some suggestion that declines in the proportion of higher order births in India may also play an explanatory role (Bhat 2002; Guilmoto 2009).

There is some work exploring the association of sex composition of children and other demographic factors with SRB in India. Bhat and Zavier (2007) found that women with no previous male births were more likely to have a male birth than women who already had a previous male birth. The distortion in the SRB is particularly pronounced in higher order births, when only daughters were born previously (Bhat 2002; Bhat and Zavier 2003; Chaudhuri 2012; Jayaraj 1999; Retherford and Roy 2003). In India, the dual desire for small families and male children puts considerable pressure on sonless couples to sex select at lower parities (DasGupta and Bhat 1997; Guilmoto 2009). Jayachandran (2017) suggests that fertility decline could explain up to half of India's child sex ratio increase during 1981–2011. Kashyap and Villavicencio (2017), using agent‐based modeling of scenarios representing South Korea and India, reported that even low levels of son preference can cause significant SRB distortions when technology diffuses steadily and fertility falls quickly. They reported that, in India, the SRB rise was less steep than South Korea due to slower diffusion of technology and higher fertility levels. A missing link in this ongoing debate is research into variations in SRB by birth order, a factor that is likely to be particularly important in societies affected by son preference, as in the case in India. A hospital‐based prospective study from Delhi, based on 2773 mothers, reported an SRB of 124 males per 100 female births; the SRB for primiparous mothers was 115. While the SRB for mothers with one living daughter was 139, the SRB for mothers with one living son was 98. The SRB for mothers with two living daughters was even more distorted (Manchanda et al. 2011). Notably the estimates of SRB in Manchanda et al. (2011) are based on small sample sizes and may not be relied upon. For example, at birth order 1, the estimates are based on 455–499 mothers. At birth order 2, the estimates of SRB are based on just 99 mothers.

There is also some research exploring the associations of socioeconomic and residence‐related factors with the SRB. Studies from Guilmoto and Ren (2011) and Jha et al. (2011) show a strong positive association between socioeconomic status and SRB in China and India, respectively. Agnihotri (2003) and Siddhanta and Nandy (2003) also found a positive association between the level of expenditure and male births in India. A geostatistical analysis by Guilmoto (2008), using 2001 Indian census district‐level data, found district‐level economic development to be one of the major correlates of the SRB. While Echavarri and Ezcurra (2010) found an inverted U‐shaped relationship between education and SRB in India, Bhat and Zavier (2007) found no consistent associations between standard of living, urban residence, educational level, religion, and caste or tribe with the SRB. Kaur et al. (2016) reported improvement in SRB in favor of females with an increase in female education. Maternal age was associated with the SRB in 1992–1993 but not in 1998–1999 (Bhat and Zavier 2007), highlighting that observed associations may be dynamic. Clark (2000) found that couples who are less educated, live in rural areas, do not belong to the scheduled castes (SCs), are Hindu or Muslim, or live in northern India not only want but also have the highest proportion of sons. Finally, regional comparisons within India found that, compared with the south, the north region had a higher SRB even after adjusting for several socioeconomic and demographic factors (Arokiasamy 2007; Bhat and Zavier 2007; Bhat 2002; Chao and Yadav 2019; Guilmoto 2008; Sudha and Rajan 1999).

Kinship structures are often used effectively in demographic research (Chakraborty and Kim 2010; Dyson and Moore 1983; Todd 1985) as they are key components of gender arrangements in a population (Dube 1997; Kaser 2008). Traditional kinship structures play a major role in determining the bargaining power of women in developing countries like India (Agarwal 1994, 1997; Folbre 1997; Miller 1981). For example, women's bargaining position is likely to be lower in societies where cross‐cousin marriages are not allowed or where child marriages are encouraged and supported (Agarwala 1957; Agarwal 1994; Mathur 2007). Studies have also shown that while a nuclear household may lack support systems, it can offer greater freedom to individual household members. There is more financial and mobility freedom for woman in nuclear households compared with non‐nuclear households. Greater exposure to household finances and mobility may lead to greater awareness and knowledge of life options among women from nuclear households compared with their counterparts from non‐nuclear households (Larsen et al. 2005). Relatedly and importantly, mothers‐in‐law were found to have an important influence on family decisions pertaining to activities within the household in rural Madhya Pradesh (Char et al. 2010), rural Bihar (Kumar, Bordone and Muttarak 2016), and urban Uttar Pradesh (Speizer et al. 2015), India. Mothers‐in‐law were also likely to influence the number of sons their daughters‐in‐law had, and the timing of their daughters‐in‐law being sterilized (Bhat and Zavier 2003; Char et al. 2010). Studies relating kinship structure and the SRB reveal mixed findings. Although a lower SRB was observed among women who were married to close relatives compared with women married to nonrelatives in a study by Jayaraj (1999), the SRB was not associated with consanguinity in marriage in a study by Bhat and Zavier (2007).

Key gender‐related characteristics that are often less examined for want of data are household patriarchal customs and norms. A few studies from Southeast Asia have identified landholding size of households as a proxy of patriarchal customs and norms (Arokiasamy and Goli 2012; Banister 2004). Bagchi (1981) stated that land is the most valuable economic resource through which men continue to determine power dynamics within genders even in the modern age. Moreover, with India being a predominantly agrarian society, land ownership is an important property right (Agarwal 1994; Government of India and UNDP 2008; Valera et al. 2018). Landholding households might prefer sons over daughters either to protect the land or to ensure inheritance of the land to sons. A study by Arokiasamy and Goli (2012) found a positive association between landholding size and CSR in rural India, with land ownership associated with having more boys than girls, highlighting the need for the consideration of this variable in the sex ratio discourse. Guilmoto and Ren (2011) also found a considerably higher SRB in mothers engaged in land‐based occupations in China.

Although prior research offers important insights, a majority of existing studies are based on macro‐analysis of census data, which contain limited variables with which to explore factors associated with SRB. Only one study focused specifically on understanding factors associated with the SRB in India using nationally representative microdata (Bhat and Zavier 2007). Bhat and Zavier (2007) examined data from the 1992–1993 and 1998–1999 National Family Health Surveys (NFHS) to assess factors associated with the SRB in India, but a number of important factors (e.g., landholding, kinship structure and community level fertility) were not included. Moreover, that study is more than a decade old. India has undergone tremendous socioeconomic and structural transformations and substantial decline in fertility in the last two decades. Interestingly, the access to modern technologies that may be used to detect the sex of fetus has also increased considerably in the last two decades. Hence, this paper examines factors associated with the SRB in India using microdata from the most recent NFHS, conducted in 2015–2016. We focus on births occurring between 2005 and 2016 to examine recent associations, and consider socioeconomic and residence‐related factors as well as kinship structure. Additionally, births more than 10 years in the past are highly masculine because of a likelihood of omission of daughters born well before the survey (United Nations Population Fund 2020). Our study will allow us to evaluate whether some of these associations have changed over the last 15 years. Additionally, we stratify analyses by birth order, an approach that can inform any changes in patterns of sex selection for sons by birth order in the recent past.

## DATA AND METHODS

### Data

Our analysis is based on 553,461 births occurring between 2005 and 2016 and reported in households interviewed as part of NFHS‐4. NFHS‐4 is a nationally representative household survey conducted across the 29 states and 6 union territories of India. The key objectives of NFHS‐4 are to provide state‐ and district‐level estimates of fertility, mortality, family planning, maternal and child health indicators, and related factors. The household and individual response rates in NFHS‐4 were 98 percent and 97 percent, respectively. Since NFHS‐4 follows a complex survey design, the estimates are representative only after weighting. The details of NFHS‐4 can be found elsewhere (IIPS and ICF 2017).

Quality of data matters a great deal for measuring SRB. NFHS follows standardized sampling design, training, and field protocols to ensure that the data collected are of optimal quality. NFHS follows a two‐stage sampling design in both urban and rural areas. In rural areas, villages are selected in the first stage using a probability proportional to size (PPS) scheme. Households are selected in second stage using systematic sampling. In urban areas, census enumeration blocks are selected in first stage using PPS scheme and households are selected in second stage using systematic sampling. Studies have shown that data collected in NFHS are of reasonably good quality (Bhat 1995; Clark 2000; Rajan and James 2008; Roy and Chattopadhyay 2012; United Nations Population Fund 2020). Moreover, the NFHS‐4 SRB estimates for the period 2004–2010 are very close to SRS and Health Management Information System (HMIS) estimates (United Nations Population Fund 2020). A recent study also suggests that nonsampling errors in NFHS‐3 and NFHS‐4 are limited only to sensitive questions such as sexual behavior, domestic violence, wife justifying domestic violence, and so on (Singh et al. 2020).

### Variables

The details of the variables included in the analysis are shown in Table 1. The demographic variables included in the analysis are birth order of the child, the existence of a surviving male sibling, and mother's age at birth of index child. We also included a variable related to the difference between the ideal number of sons and daughters, dichotomized into “equal numbers or more daughters” versus “more sons.” We also estimated the average community fertility, operationalized as the number of children per woman at the primary sampling unit (PSU) level. Note that PSUs in rural and urban areas are villages and census enumeration blocks, respectively (IIPS and ICF 2017).

**TABLE 1:** SRB by various socioeconomic, demographic, residence‐related, and kinship structure‐related variables, India 2005–2016

					95% CI for SRB	
Characteristics	Categories	Distribution of births (%)	Number of births	SRB (M/F)	Lower	Upper
Demographic characteristics						
Birth order	First	36.3	200,959	107.5	106.5	108.4
	Second	30.6	169,320	108.3	107.3	109.4
	Third or higher	33.1	183,182	112.3	111.3	113.3
Surviving male sibling and birth order 2 or higher	No	62.0	343,286	111.4	110.7	112.1
	Yes	38.0	210,174	105.8	104.9	106.5
Difference between ideal number of sons and daughters	Equal numbers or more daughters	75.2	416,023	103.3	102.7	103.9
	More sons	24.8	137,438	130.4	129.0	131.8
Mother's age at birth of index child	<20 years	1.3	7,208	106.2	101.4	111.2
	20–29 years	51.3	283,719	108.3	107.5	109.1
	30+ years	47.4	262,535	110.5	109.7	111.3
Average community fertility (per woman)	>2.8	8.2	45,389	103.7	101.8	105.6
	>2.1 and 2.8≥	31.2	172,820	108.3	107.3	109.3
	>1.5 and 2.1≥	43.3	239,817	110.5	109.6	111.4
	≤1.5	17.2	95,434	111.9	110.5	113.3
Socioeconomic characteristics						
Mother's schooling	No schooling	36.3	201,058	107.5	106.5	108.4
	Primary	14.7	81,481	109.6	108.1	111.2
	Secondary	40.6	224,723	111.0	110.1	111.9
	Higher	8.3	46,198	111.0	109.0	113.0
Wealth index	Poorest	26.5	146,656	105.8	104.7	106.9
	Poorer	21.8	120,819	107.9	106.7	109.1
	Middle	19.4	107,560	111.0	109.7	112.3
	Richer	17.6	97,245	110.1	108.7	111.5
	Richest	14.7	81,180	116.0	114.4	117.6
Caste	ST	10.6	58,635	104.5	102.8	106.2
	SC	21.4	118,666	108.3	107.1	109.6
	OBC	44.1	243,971	110.1	109.2	111.0
	Other	23.9	132,189	111.4	110.2	112.6
Religion	Hindu	78.6	434,784	109.6	109.0	110.3
	Muslim	16.5	91,481	108.3	106.9	109.7
	Other	4.9	27,196	110.1	107.5	112.8
Land holding size of the household	No land	58.4	323,471	108.8	108.1	109.6
	Up to 10 acres	8.1	44,608	109.6	107.6	111.7
	More than 10 acres	17.1	94,608	111.9	110.5	113.3
	Land unit not defined	16.4	90,774	108.8	107.4	110.2
Residence‐related characteristics						
Urban–rural residence	Urban	28.4	156,964	111.4	110.3	112.5
	Rural	71.6	396,497	108.8	108.1	109.4
State–regions	South	17.3	95,601	107.5	106.1	108.8
	North	13.9	76,915	116.0	114.4	117.6
	Central	27.5	152,113	109.2	108.1	110.3
	East	25.1	138,799	107.0	105.9	108.2
	Northeast	3.7	20,676	105.8	102.9	108.7
	West	12.5	69,357	111.4	109.8	113.1
Kinship structure‐related characteristics						
Household structure	Nuclear	48.4	267,644	107.9	107.1	108.7
	Non‐nuclear	51.6	285,817	111.0	110.2	111.8
Presence of an elderly woman (age 60+) in the household	No	78.8	436,383	108.8	108.2	109.4
	Yes	21.2	117,078	112.3	111.0	113.6
Consanguineous marriage	No	86.6	479,201	109.2	108.6	109.8
	Yes	13.4	74,260	111.0	109.4	112.6
Child marriage	No	53.1	293,387	110.1	109.2	110.8
	Yes	46.9	259,635	108.8	108.0	109.6
Total			553,461	109.2	108.6	109.8

The socioeconomic variables included in the statistical analysis are mother's schooling, wealth index, caste, religion, and landholding size of households. The wealth index, already included in the NFHS‐4 dataset, is a principal component analysis‐derived index of household assets and amenities (IIPS and ICF 2017). Caste is coded into four categories namely “SCs,” “scheduled tribes (STs),” “other backward class (OBC),” and other. The former three groups are considered socially and economically marginalized by the Constitution of India, and hence receive various protections and support services. SC and ST are the most heavily disenfranchised of the socioeconomic strata, followed by the OBC (Bhagat 2011). They have different levels of deprivation (IIPS and ICF 2017), and hence are differentially identified in the Indian Constitution. In India, Hindus are the most numerous religious group followed by Muslims and Christians. There are smaller religious groups like Sikhs, Jains, Buddhists/neo‐Buddhists, and others (Office of the Registrar General of India 2011). Based on sample size, religion was categorized as Hindu, Muslim, and other. We aggregated land holding size of the household into four categories—no land, up to 10 acres, more than 10 acres, and land unit not defined (i.e., land for which the unit of measurement was not recorded in the survey).

Residence‐related characteristics included urban–rural residence and state–regions, namely, North, Central, East, Northeast West, and South (IIPS and ICF 2017). The states included in the six geographic regions of India are shown in Table A1 in the Supporting Information.^1^

We included four variables—consanguineous marriage, child marriage, household structure, and presence of an elderly women (60+) in the household—to account for the role of kinship structure in determining the SRB in India. Consanguineous marriage is defined as a marriage where the (current) husband was related to the wife in any way before she got married, coded as no/yes. Child marriage is defined as a marriage/cohabitation (whichever is earlier) initiated when the woman was younger than age 18. Household structure is coded as nuclear versus non‐nuclear. To account for the influence of mothers‐in‐law or mothers, we constructed a proxy variable assessing the presence of an elderly woman (60+) in the household.

### Analytic Methods

We fit multivariable logistic regression models to examine the association between the variables listed in Table 1 and the probability of a male birth, with separate models used for birth orders 1, 2, and 3 or higher. We used “svy” commands available in STATA 16.0 for adjusting our estimates for the complex survey design and weights used in NFHS‐4. The SRB in this paper is defined as male births per 100 female births. If 106 (i.e., biological normal upper limit for SRB) lay within the 95 percent CI of SRB for a category of covariate, then the SRB for that category of covariate was not considered significantly different from the biological upper limit.

## RESULTS

### Descriptive Results

#### SRB by Birth Order, Sex Composition of Children, Desired Ideal Number of Sons and Daughters, and Fertility in the Immediate Community

The SRB generally increased with an increase in birth order, ranging from 107.5 for birth order 1 to 112.3 for birth orders 3 or higher (Table 1). The SRB at birth order 1 is above the biological normal for first births in India, and was significantly different from the SRB at birth orders 3 or higher. The SRB in the absence of a surviving male sibling was 111.4, which is far above the biological normal level, and significantly different from the SRB in the presence of a surviving male sibling (105.8, which is within normal limits). The SRB also varied considerably between mothers who desired an equal number of sons and daughters as ideal or more daughters than sons as ideal (103.3, within normal limits), and mothers who desired more sons than daughters as ideal (130.4). In our study, the SRB is well within the normal range when community‐level fertility is above 2.8 children per woman (103.7). It jumps to 111.9 among mothers in communities where average fertility is 1.5 children per woman or lower.

#### SRB by Socioeconomic Characteristics

Table 1 also shows the SRB by selected socioeconomic, residence‐related, and kinship structure‐related variables. The SRB increased with an increase in mother's schooling; it was significantly lower among mothers with no schooling compared with mothers having completed secondary schooling. The SRB varied considerably by household wealth, with a statistically significant difference between the lowest levels (among the poorest quintile [105.8]) and highest levels (among the richest quintile [116.0]). The SRB is lowest among ST followed by SC, OBC, and other. The SRB among mothers in households with landholding size more than 10 acres was 111.9, whereas it was 108.8 among mothers in households having no landholding.

#### SRB by Residence‐Related Characteristics

We found higher SRB in urban areas (111.4) than in rural (108.8) areas. In terms of geographic regions, the SRB was highest in the north (116.0) and lowest in the northeast (105.8). The SRB was also elevated in the west (111.4) and the central regions (109.2). Interestingly, the south, which is not known for sex selection, has a SRB that is outside of the normal range (107.5).

#### SRB by Kinship Structure‐Related Characteristics

Of the four kinship structure‐related variables, only household structure and presence of an elderly woman in the household were associated with the SRB in the bivariate analysis. The SRB was lower in nuclear households (107.9) compared with non‐nuclear households (111.0). Likewise, the SRB was lower in households without an elderly woman (108.8) compared with households with an elderly woman (112.3).

### Multivariable Regression Results Stratified by Birth Order

Multivariable regression results at the national level are presented in Table 2. Since the SRB varies considerably by birth order, we estimated separate logistic regressions for birth order 1, birth order 2, and birth orders 3 or higher. If the SRB at birth order 1 in India is random, it should be responsive to a few socioeconomic status variables because only the upper socioeconomic classes would care to sex‐select for the first pregnancy. The regression results for birth order 1 suggest that the probability of a male birth is indeed responsive to a few variables indicative of economic status and son preference. The probability of a male birth was associated with higher wealth quintiles ‐ mothers from the middle and richest quintiles were more likely to have a male birth than mothers from the poorest quintiles. Likewise, the odds of having a male as opposed to female birth was higher among mothers who desired more sons than daughters (adjusted odds ratio [AOR] = 1.54; 95 percent confidence interval [CI]: 1.49, 1.59) than among mothers who did not report preferring more sons. The odds of having a male birth was also higher among mothers in communities where the average number of children per woman was less than or equal to 2.1 than among mothers in communities where the average is greater than 2.8. In other words, women in communities with lower relative to higher fertility were more likely to have a male birth. We also found that mothers from the central and the east regions were less likely to have a male birth than mothers from the south.

**TABLE 2:** Results of multivariable logistic regression analysis of determinants of having a male birth, based on births between 2005 and 2016, India

		Odds ratio (95% CI)		
Characteristics	Categories	Birth order 1 (1)	Birth order 2 (2)	Birth order 3 or higher (3)
Demographic characteristics				
Surviving male sibling and birth order 2 or higher	Yes®			
	No	NA	1.13 (1.09, 1.16)*	1.14 (1.11, 1.17)*
Difference between ideal number of sons and daughters	Equal numbers or more daughters®			
	More sons	1.54 (1.49, 1.59)*	1.34 (1.29, 1.39)*	1.18 (1.15, 1.21)*
Mother's age at birth of index child	<20 years®			
	20–29 years	0.99 (0.91, 1.07)	1.10 (0.90, 1.33)	1.38 (0.70, 2.71)
	30+ years	0.97 (0.88, 1.06)	1.11 (0.91, 1.35)	1.42 (0.72, 2.80)
Average community fertility (per woman)	>2.8®			
	>2.1 and 2.8≥	1.02 (0.97, 1.08)	1.05 (1.00, 1.10)	1.06 (1.02, 1.09)*
	>1.5 and 2.1≥	1.09 (1.03, 1.15)*	1.09 (1.04, 1.15)*	1.06 (1.02, 1.10)*
	≤1.5	1.10 (1.03, 1.17)*	1.11 (1.05, 1.19)*	1.13 (1.06, 1.20)*
Socioeconomic characteristics				
Mother's schooling	No schooling®			
	Primary	1.00 (0.96, 1.04)	1.02 (0.98, 1.07)	1.04 (1.00, 1.07)*
	Secondary	1.02 (0.98, 1.06)	1.02 (0.98, 1.05)	1.07 (1.04, 1.11)*
	Higher	1.00 (0.95, 1.06)	1.00 (0.93, 1.07)	1.25 (1.13, 1.38)*
Wealth index	Poorest®			
	Poorer	1.01 (0.97, 1.05)	1.01 (0.97, 1.05)	1.02 (0.99, 1.05)
	Middle	1.05 (1.00, 1.09)*	1.04 (0.99, 1.08)	1.04 (1.01, 1.09)*
	Richer	1.01 (0.96, 1.06)	1.02 (0.97, 1.08)	1.11 (1.05, 1.16)*
	Richest	1.07 (1.01, 1.14)*	1.10 (1.03, 1.17)*	1.14 (1.06, 1.22)*
Caste	Other®			
	ST	0.98 (0.93, 1.03)	0.94 (0.89, 1.00)*	0.92 (0.87, 0.96)*
	SC	1.03 (0.98, 1.07)	0.95 (0.90, 0.99)*	0.95 (0.91, 0.99)*
	OBC	1.02 (0.98, 1.05)	0.97 (0.93, 1.00)	0.97 (0.94, 1.01)
Religion	Hindu®			
	Muslim	1.00 (0.96, 1.04)	0.96 (0.91, 1.00)*	0.96 (0.93, 0.99)*
	Other	1.02 (0.96, 1.08)	0.98 (0.92, 1.05)	1.00 (0.93, 1.07)
Land holding size of the household	No land®			
	Up to 10 acres	0.99 (0.95, 1.04)	1.04 (0.99, 1.09)	1.01 (0.97, 1.06)
	More than 10 acres	0.98 (0.95, 1.02)	1.06 (1.02, 1.11)*	1.04 (1.00, 1.08)*
	Land unit not defined	1.00 (0.96, 1.04)	1.00 (0.96, 1.04)	0.98 (0.95, 1.01)
Residence‐related characteristics				
Urban–rural residence	Urban®			
	Rural	0.99 (0.96, 1.03)	1.03 (0.99, 1.07)	0.99 (0.95, 1.03)
State–regions	South®			
	North	1.04 (0.99, 1.09)	1.08 (1.03, 1.13)*	1.13 (1.06, 1.20)*
	Central	0.94 (0.90, 0.99)*	1.05 (1.00, 1.10)*	1.08 (1.02, 1.15)*
	East	0.94 (0.89, 0.99)*	1.04 (0.99, 1.09)	1.10 (1.04, 1.17)*
	Northeast	1.02 (0.96, 1.08)	0.98 (0.92, 1.04)	1.08 (1.00, 1.16)*
	West	0.99 (0.93, 1.05)	1.05 (0.98, 1.11)	1.16 (1.07, 1.25)*
Kinship structure‐related characteristics				
Household structure	Nuclear®			
	Non‐nuclear	1.01 (0.98, 1.05)	1.01 (0.98, 1.05)	1.00 (0.97, 1.04)
Presence of an elderly woman (age 60+) in the household	No®			
	Yes	1.01 (0.98, 1.05)	1.02 (0.98, 1.07)	1.01 (0.97, 1.04)
Consanguineous marriage	No®			
	Yes	0.99 (0.94, 1.03)	1.06 (1.01, 1.11)*	1.04 (1.00, 1.08)
Child marriage	No®			
	Yes	1.01 (0.98, 1.05)	0.97 (0.94, 1.00)	1.00 (0.98, 1.03)

* p < 0.05. ®Shows reference category.

NOTE: Results additionally adjusted for year of birth. NA, not applicable.

As with birth order 1, a limited number of variables were associated with the probability of a male birth at birth order 2. The absence of a living male sibling was associated with a higher probability of male birth. The odds of a male birth were higher among mothers who had no living son (AOR = 1.13; 95 percent CI: 1.09, 1.16) than among mothers who already had a son. Mothers who desired higher ideal number of sons than daughters had higher odds of a male birth (AOR = 1.34; 95 percent CI: 1.29, 1.39) than did their counterparts who did not report a preference for more sons. The effect of average fertility at the community level became stronger at birth order 2. The odds of a male birth were 10 percent higher (AOR = 1.10; 95 percent CI: 1.03, 1.17) among mothers from the richest household wealth quintile than among mothers from poorest quintile. Mothers from ST and SC were significantly less likely to have a male birth compared with mothers who were not from a ST, SC, or OBC. Likewise, Muslim mothers were less likely to have a male birth compared with Hindu mothers. Interestingly, mothers from households having 10 acres or more land had higher odds of a male birth (AOR =1.06; 95 percent CI: 1.02, 1.11) than did mothers from households that do not own land. We also found that mothers from the north and central regions were more likely to have a male birth than mothers from the south.

Most SRB correlates are visible at birth orders 3 or higher. The absence of a living male sibling (odds ratio: 1.14; 95 percent CI: 1.11, 1.17) and the difference in the desired ideal number of sons and daughters (odds ratio: 1.18; 95 percent CI: 1.15, 1.21) were positively associated with the probability of a male birth. Average fertility at the community level was also associated with the probability of a male birth. Mothers in communities where the average number of children per woman was between 2.2 and 2.8 were 1.06 (95 percent CI: 1.02, 1.09) times as likely as having a male birth compared with mothers in communities where the average number of children per woman is greater than 2.8. Likewise, mothers in communities where the average number of children per woman was between 1.6 and 2.1 were 1.06 (95 percent CI: 1.02, 1.10) times as likely as having a male birth compared with mothers in communities where the average number of children per woman is greater than 2.8. Similarly, mothers in communities where the average number of children per woman was less than 1.6 were 1.13 (95 percent CI: 1.06, 1.20) times as likely as having a male birth compared with mothers in communities where the average number of children per woman is greater than 2.8.The probability of a male child was also associated with mother's schooling. The odds of a male birth were 25 percent higher (AOR = 1.25; 95 percent CI: 1.13, 1.38) among mothers having schooling higher than secondary, and 7 percent higher among mothers with secondary schooling (AOR = 1.07; 95 percent CI: 1.04, 1.11) than among mothers with no schooling. Mothers from the middle (AOR = 1.04; 95 percent CI: 1.01, 1.09), richer (AOR = 1.11; 95 percent CI: 1.05, 1.16), and richest (AOR = 1.14; 95 percent CI: 1.06, 1.22) quintiles were more likely to have a male birth than mothers from the poorest quintile. Mothers from ST and SC were less likely to have a male birth compared with mothers who are not from a ST, SC, or OBC. Muslim mothers were less likely than Hindu mothers to have a male birth (AOR = 0.96; 95 percent CI: 0.93, 0.99). Mothers from the north, central, east, northeast, and west regions were more likely to have a male birth than mothers from the south. The landholding size of the household was also associated with SRB at birth orders 3 or higher (AOR = 1.04; 95 percent CI: 1.00, 1.08).

## DISCUSSION

Our study provides robust estimates of the SRB in India by various socioeconomic, demographic, residence‐related, and the kinship‐related variables. Importantly, we also provide 95 percent CIs for the estimated SRBs in India, facilitating comparison across groups. We estimate a national SRB of 109.2 for births occurring between 2005 and 2016. This is comparable to SRBs reported in other studies, including the Indian SRS (SRB of 110.7 for 2005–2015) and the District Level Household Survey 2007–2008 (SRB of 109.6 for 2004–2008) (IIPS 2010). While the 2001 Indian Census provided an estimate of 110.4, the 2011 Indian Census provided an estimate of 111.2 (both based on births during the preceding year) (Rajan et al. 2017). Overall, these findings demonstrate that the SRB remains a major concern in India, even as fertility rates have fallen from 2.9 children per woman in 2005 to 2.2 children per woman in 2018 (Office of the Registrar General of India 2006; Office of the Registrar General of India 2020).

Such high levels of the SRB have also been observed in other south Asian countries marked by considerable son preference. For example, the SRB for China from the 2005 Intercensal Survey was 120.5, and the Official Estimate for 2009 was 119.5. The first 2010 census estimate is 118.1 (Li, 2007; Li et al., 2007; NSB, 2010). The SRB in Vietnam also reached as high as 113.8 in 2013 (General Statistics Office of Vietnam 2013). In South Korea, the SRB declined from as high as 116.5 in 1990 to 106.2 in 2007 (Boer and Hudson 2017).

There is generally an absence of SRB data stratified across birth orders in India. The importance of filling this gap is demonstrated in our study, in which SRB varied considerably by birth order. Although the SRB was highest for third or higher order births (112.3), even the SRB at birth order 1 (107.5) exceeded the normal range. The only study from India that provides a comparable estimate of the SRB at birth order 1 uses data from 1998‐1999 to show a SRB of 106.9 at birth order 1, suggesting almost no change in the SRB at birth order 1 in India in almost 20 years (Bhat and Zavier 2007). Our estimates of SRB at birth order 1 are comparable with similar estimates from other south Asian countries that are marked by considerable son preference, including China and Vietnam (Boer and Hudson 2017; Guilmoto and Ren 2011).

There is a lack of data on factors influencing SRB by birth order in India. Our analysis indeed suggests that the factors influencing male births are different at different birth orders. We found that the SRB at birth order 1 was responsive to a few socioeconomic variables. At the national level, the probability of a male birth was associated with being in the middle and richest wealth quintiles, residing in a lower fertility community, and living in the central and east regions. Interestingly, most of these SRB correlates were visible for birth order 3 or higher.

Mothers who had no living son were more likely than mothers who had a son to have a male birth. This finding is consistent with the findings of previous studies in India (Bhat 2002; Bhat and Zavier 2003; Bhat and Zavier 2007; Jayaraj 1999; Retherford and Roy 2003). Mothers who desired a higher number of sons than daughters as ideal are more likely to have a male birth than mothers who reported other combinations. Notably, the effect of this son preference indicator, while seen across birth order, was largest for first order births, and was larger for second order births than for third and higher births. These findings suggest that son preference remains a key driver of SRB in India.

Our study also highlights the role played by community fertility in distorting SRB in favor of males in India. Lower fertility levels in the immediate community were associated with a higher probability of a male birth at all the birth orders. Women in low fertility communities were more likely than women in high fertility communities to have a male birth. This finding is consistent with that of Jayachandran (2017), who reported that up to half of India's child sex ratio increase during 1981–2011 could be explained by fertility decline. As efforts are made to increase contraceptive access and use in India, we may continue to see these effects across more of the population, unless more effort is made to tackle social norms that reinforce son preference in the country.

A key area that likely affects son preference and SRB is land inheritance, which despite legal changes continues to favor sons over daughters (Bhalotra et al. 2017; Deininger et al. 2013). Our study offers evidence in support of the landowning patriarchy hypothesis, finding that at birth orders 2 and 3 or higher, mothers from households holding 10 or more acres of land were more likely to have a male birth than mothers from households having no land. Recognizing that landholding size is more meaningful in rural areas, we ran an additional exploratory analysis in which the main regression models were run for rural areas only. Our results were similar to those presented in Table 2 (results not shown). Our findings are also in line with studies from China where the SRB was relatively high for mothers engaged in a land‐based occupation (Guilmoto and Ren 2011). Moreover, land reforms accounted for roughly half of the increase in sex ratios in rural China from 1978–1986 (Almond et al. 2019). Banister (2004) also argued for carefully understanding the system of landholding and transmitting land and property rights within families to explain shortage of girls in China, lessons that may be directly relevant to the many ways landholding may affect the SRB in India. While Arokiasamy and Goli (2012) argue that daughters have weak ties with their natal families after marriage due to restriction of land rights to males of the patrilineal clan, Guilmoto and Ren (2011) suggest that women are economically less valued in landed households. Arokiasamy and Goli (2012) also reported lower female autonomy in large landholding households. In India, it has been the sons who inherit land, and parents largely depend on their sons for old‐age support. Norms that reinforce male inheritance of property and property management, including views that girls leave the family at marriage and women cannot take responsibility for finances and land, serve to sustain the greater desire for and value of boys in land owning households.

South India, which is not known for sex‐selection, had a SRB of 107.5. While a SRB of 107.5 seems high for the south, it is confirmed by other sources including the Census of India and the SRS. The Census of India indicates that the SRB at birth in the south increased from 106 in 2001 to 107 in 2011. The SRS provides a SRB estimate of 107.4 for the period 2005–2016. Result adjusted for average fertility at the community level and other variables also suggests that the SRB (109.0; 95 percent CI: 108.7, 109.3) in the south is outside the biological normal limit. This evidence indicates that, despite the popular assumption that son preference is not a concern in the south; couples in this region are also adopting sex selection to have sons, although to a much smaller extent than seen in other regions of India.

A comparison of our results with Bhat and Zavier (2007) suggests some changes in the last two decades. Although birth order, educational level, standard of living, and caste failed to show significant or consistent relationship with the SRB in Bhat and Zavier (2007), these were significantly and consistently associated with the SRB in our study (Table A2 in the Supporting Information). In our study, the probability of a male birth consistently increased with increase in birth order, mother's schooling, and household wealth. The probability of a male birth was lower among ST compared with others. Although Bhat and Zavier (2007) found a spurious nonlinear relationship between desired ideal number of children and the SRB, we found a consistent relationship between desired higher number of sons than daughters as ideal and higher probability of a male birth.

### Strengths and Limitations of the Study

A key strength of our study is the use of over 553,000 births occurring between 2005 and 2016 in representative households to study the factors associated with SRB in India. Due to the micro nature of data, we were able to include a number of important variables including kinship‐structure related variables, landholding size of households, and the average fertility at the community level into the regression models. This study also has some important limitations. First, we could not account for genetic factors and under‐reporting of female children (if any) due to unavailability of data. There is a possibility in societies highly affected by son preference that the mother reports her son(s) first and then the daughter(s), affecting our estimates. However, this risk is lessened with the NFHS‐4 questionnaire, in which mothers were asked to list all births in the order in which they occurred starting with her first birth. Second, we could not include certain variables like women's work and father's characteristics, as in NFHS‐4, this information was only collected in 15 percent of the randomly selected households. Third, although we controlled for state‐regions in regressions, we could not fully capture the cultural practices and norms regarding son preference or daughter aversion, level of patriarchy in different geographical regions of India due to nonavailability of data in NFHS‐4. Finally, since the SRB is highly affected by sample size, any analysis of SRBs estimated from survey data should be interpreted with caution. For example, for an observed sex ratio of 107, the 95 percent CI is 94.5, 121.2 if the estimation is based on a sample of 1,000 births, 101.2, 113.1 if based on a sample of 5,000 births, 102.9, 111.3 if based on a sample of 10,000 births, and 105.7, 108.3 if based on a sample of 100,000 births, even for samples derived without considering clustering. The confidence intervals are likely to be even wider for large‐scale sample surveys, which are largely multistage cluster samples. Fortunately, our analysis is based on very large samples, thus providing robust estimates of the SRB at the national level.

## CONCLUSION

Our in‐depth analysis of diverse factors associated with the SRB across birth orders in India offers several key insights. The SRB at birth order 1 is above the normal upper limit for the country as a whole and across national regions, which is a matter of great concern and requires further explanatory research. Lower levels of community fertility were associated with higher SRB for all birth orders, underlining the role of fertility decline in distorting SRB in favor of males. Our findings also call for more debate around the landholding‐patriarchy hypothesis to formulate policies and programs that directly address son preference and gender‐based discrimination. Implementing equal land rights law and restricting the male dominance in ownership and inheritance of land may be inadequate in the absence of policies and programs to change cultural norms regarding patriarchy and patrilocality. In absence of such policies, implementation of equal land rights law may actually exacerbate son preference in India (Bhalotra et al. 2017). Given that fertility is declining in India and that sex selection is spreading to societies that were not previously known for sex selection such as south India, there is an urgent need for regular monitoring of the SRB. The Government of India in the recent past has launched several programs, including but not limited to “Beti Bachao and Beti Padhao,” to protect the girl child from discrimination before and after her birth. Ongoing monitoring of changing trends in SRB and the social norms that reinforce it are critical to understanding the impact and adequacy of Government's current initiatives to improve the value and status of women and girls and correct skewed SRBs in India.

## ETHICS APPROVAL STATEMENT

Our analysis is based on 553,461 births occurring between 2005 and 2016 in households interviewed in NFHS‐4. The NFHS‐4 is a publically available dataset with no individual identifiers in place. Hence, our study is exempt from ethical approval.

## Supporting information

Supporting Information

## Data Availability

Our analysis is based on 553,461 births occurring between 2005 and 2016 in households interviewed in NFHS‐4. The NFHS‐4 is a publicly available dataset with no individual identifiers in place. The NFHS‐4 dataset may be freely accessed from the DHS Program website: https://dhsprogram.com.

## Footnotes

1 Appendixes are available at the supporting information tab at wileyonlinelibrary.com/journal/sfp.
