# Supplementary material for: Factors Influencing the Sex Ratio at Birth in India: A New Analysis based on Births Occurring between 2005 and 2016
Source: Stud Fam Plann. 2021 Feb 22;52(1):41–58. doi: 10.1111/sifp.12147 (PMC8049007; doi:10.1111/sifp.12147)
Supplement: Supplementary file 1 — Supporting Information [file SIFP-52-41-s001.docx]

**Appendix T1**

**List of states included in the six geographic regions of India**

| **State-regions** | **States** |
| --- | --- |
| North | Chandigarh, Delhi, Haryana, Himachal Pradesh, Jammu & Kashmir, Punjab, Rajasthan, and Uttarakhand |
| Central | Uttar Pradesh, Madhya Pradesh, and Chhattisgarh |
| East | Bihar, Jharkhand, Odisha, West Bengal |
| Northeast | Arunachal Pradesh, Assam, Manipur, Meghalaya, Mizoram, Nagaland, Sikkim, and Tripura |
| West | Dadra & Nagar Haveli, Daman & Diu, Gujarat, Maharashtra, and Goa |
| South | Andaman & Nicobar Islands, Andhra Pradesh, Karnataka, Kerala, Lakshadweep, Puducherry, Tamil Nadu, and Telangana |

**Appendix T2**

**Results of multivariable logistic regression analysis of determinants of having a male birth, based on births between 2005 and 2016, India**

| **Characteristics** | **Categories** | **OR (95% CI)** |
| --- | --- | --- |
| **Demographic characteristics** | | |
| Birth order | Third or higher® |  |
|  | First | 0.86 (0.84, 0.89)* |
|  | Second | 0.93 (0.91, 0.95)* |
| Surviving male sibling and birth order 2 or higher | Yes® |  |
|  | No | 1.15 (1.12, 1.17)* |
| Difference between ideal number of sons and daughters | Equal numbers or  more daughters® |  |
|  | More sons | 1.32 (1.30, 1.35)* |
| Mother’s age at birth of index child | <20 years® |  |
|  | 20-29 years | 0.99 (0.92, 1.06) |
|  | 30+ years | 0.99 (0.92, 1.07) |
| Average community fertility (per woman) | >2.8 |  |
|  | >2.1 & 2.8≥ | 1.05 (1.02, 1.07)* |
|  | >1.5 & 2.1≥ | 1.08 (1.06, 1.11)* |
|  | ≤1.5 | 1.10 (1.06, 1.14)* |
| **Socio-economic characteristics** | | |
| Mother's schooling | No schooling® |  |
|  | Primary | 1.03 (1.01, 1.05)* |
|  | Secondary | 1.04 (1.02, 1.06)* |
|  | Higher | 1.02 (0.98, 1.06) |
| Wealth index | Poorest® |  |
|  | Poorer | 1.02 (1.00, 1.04) |
|  | Middle | 1.05 (1.02, 1.07)* |
|  | Richer | 1.04 (1.01, 1.07)* |
|  | Richest | 1.09 (1.05, 1.14)* |
| Caste | Other® |  |
|  | ST | 0.95 (0.93, 0.98)* |
|  | SC | 0.98 (0.95, 1.00) |
|  | OBC | 0.99 (0.97, 1.01) |
| Religion | Hindu® |  |
|  | Muslim | 0.98 (0.96, 1.00) |
|  | Christian | 1.00 (0.97, 1.04) |
|  | Sikh |  |
|  | Buddhist/Neo-Buddhist | 1.01 (0.98, 1.04) |
|  | Other | 1.02 (1.00, 1.05)* |
| Land holding size of the household | No land® | 0.99 (0.97, 1.01) |
|  | Up to 10 acres | 1.03 (1.01, 1.05)* |
|  | More than 10 acres | 1.04 (1.02, 1.06)* |
|  | Land unit not defined | 1.02 (0.98, 1.06) |
| **Residence-related characteristics** | | |
| Urban-rural residence | Urban® |  |
|  | Rural | 1.00 (0.98, 1.03) |
| State-regions | South® |  |
|  | North | 1.07 (1.04, 1.10)* |
|  | Central | 1.01 (0.98, 1.04) |
|  | East | 1.01 (0.98, 1.04) |
|  | North-East | 1.02 (0.98, 1.05) |
|  | West | 1.04 (1.01, 1.08)* |
| **Kinship structure-related characteristics** | | |
| Household structure | Nuclear® |  |
|  | Non-nuclear | 1.01 (0.99, 1.03) |
| Presence of an elderly woman (age 60+) in the household | No® |  |
|  | Yes | 1.01 (0.99, 1.04) |
| Consanguineous marriage | No® |  |
|  | Yes | 1.02 (1.00, 1.05) |
| Child marriage | No® |  |
|  | Yes | 1.00 (0.98, 1.01) |
| NOTE: *p < 0.05 |  |  |
| Results additionally adjusted for year of birth |  |  |
